# Evaluation of the curative effects of Bailing capsules for treating chronic obstructive pulmonary disease: A protocol for systematic review and meta-analysis

**DOI:** 10.1097/MD.0000000000025672

**Published:** 2021-06-25

**Authors:** Long Zhang, Yumiao Zhao, Junli Jia, Lisha Huang, Weili Chu, Qinfu Xu, Yanbing Sheng, Aiguo Xu

**Affiliations:** Department of Respiratory and Critical Care Medicine, The First Affiliated Hospital of Zhengzhou University, Zhengzhou, Henan Province, China.

**Keywords:** Bailing, chronic obstructive pulmonary disease, efficacy, safety

## Abstract

**Background:**

The goal of the present study is to evaluate the efficacy and safety of Bailing capsules, which is a traditional Chinese drug that can improve lung functionality when used to treat chronic obstructive pulmonary disease (COPD) patients.

**Methods:**

A comprehensive search will be performed on the following primary electronic databases: PubMed, EMBASE, Cochrane Library, Chinese National Knowledge Infrastructure, and WanFang database. A search of secondary sources includes reference lists of included studies. Two pairs of review authors will screen and scrutinize selected articles. This study will analyze continuous data as mean differences and dichotomous data as odds ratios, both with 95% confidence intervals. A sensitivity analysis will also be conducted to evaluate the stableness of the outcomes. RevMan 5.3 software was adopted to accomplish all the statistical analysis.

**Results:**

The results obtained in this research shall be published in a peer-reviewed journal.

**Conclusion:**

Based on the interpretations of the results, useful conclusions will be presented. These conclusions will offer additional insights with useful evidence to assess whether it is viable to use Bailing capsules as an effective and safety treatment option for COPD.

**Ethics and dissemination:**

The present work does not involve any humans or animals; therefore, ethical approval is not needed.

**Systematic review registration:**

March 26, 2021.osf.io/kvgbu. (https://osf.io/kvgbu/)

## 1 Introduction

Chronic obstructive pulmonary disease (COPD) is a widespread illness. It is primarily characterized by bronchial obstruction and inflammation of the chronic airway, which results in gradually impeding the airflow.^[1]^ Globally, COPD is ranked as the third leading cause of fatalities. In 2005, COPD killed over 3 million people, which accounted for 6% of all global fatalities.^[1,2]^ COPD is a critical health problem that is both avoidable and curable. Globally, COPD is a primary cause of chronic morbidity and mortality. Most patients suffer from COPD for years and pass away prematurely due to the disease itself or complications caused by COPD.^[3]^ The underlying mechanism is related to elevated chronic inflammatory response in the airways and within the lungs to toxic airborne particulates or gases.^[1]^ Clinically, continuing and advancing dyspnea is primarily characteristic of COPD. Most commonly, 30% of patients exhibited coughing with sputum generation.^[4]^ Therapeutic measures for COPD aims to alleviate symptoms, diminish aggravations, and enhance quality of life and exercise tolerance.^[4–7]^

The Bailing capsules refer to a medication that is refined by fermenting cordyceps strains at a low temperature. The cordyceps strains contain cordyceps polysaccharides and amino acids. The strains have a number of useful functionalities, which includes antioxidative properties, anti-inflammatory characteristics, proteinuria reduction ability, and effectively treating COPD.^[8,9]^ A past empirical study demonstrated that Bailing capsules have the capability to inhibit the expression of serum tumor necrosis factor-alpha and interleukin-12 in a rat model of lipopolysaccharide/interferon-induced inflammation. It was also found to have significantly increased eosinophils in the lung lavage fluid of sensitized rats’ inhibition.^[10]^ Meta-analyses indicate that Bailing capsules have better efficacy treating COPD by improving arterial blood gas index, pulmonary function index, and exercise tolerance.^[10,11]^ Still, the efficacy and safety of Bailing capsules when used to treat COPD is yet to be established. Therefore, this meta-analysis will systematically evaluate the clinical efficacy and safety of using Bailing capsule to treat COPD.

## 2 Methods

### 2.1 Registration

The present systematic review protocol is registered on the Open Science Framework (OSF) with the registration number 10.17605/OSF.IO/KVGBU. This protocol has been drafted under the guidance of the Preferred Reporting Items for Systematic Review and Meta-Analyses Protocols (PRISMA-P) statement guidelines.

### 2.2 Eligibility criteria for included studies

#### 2.2.1 Types of studies

This study employs randomized controlled trials (RCTs) to evaluate the curative effects of Bailing capsules in the treatment of COPD, regardless of blinding and allocation concealment. Reviews, case reports, conferences, non-RCTs, and studies involving animals will be excluded.

#### 2.2.2 Types of participants

This review will encompass participants who were diagnosed as COPD in accordance with the criteria of GOLD.^[1]^ Restrictions are not placed on gender, age, and course of treatment.

#### 2.2.3 Types of interventions

This review will encompass past studies comparing Bailing capsules versus routine treatment (e.g., formoterol, indacaterol, tiotropium, and aclidinium).

#### 2.2.4 Types of outcome

##### 2.2.4.1 Major outcomes

The major outcomes include trough forced expiratory volume in 1 second fluctuation from reference, St George's Respiratory Questionnaire or the Chronic Respiratory Disease Questionnaire fluctuation from reference, and subsidising of the symptoms assessed via the Transition Dyspnea Index.

##### 2.2.4.2 Minor outcomes

The minor outcomes include total serious adverse events (SAEs), pneumonia reported as SAE, and death.

### 2.3 Search methods for identification of studies

#### 2.3.1 Search strategy

This review will perform a systematic exploration in PubMed, EMBASE, Cochrane Library, Chinese National Knowledge Infrastructure, and WanFang database. These electronic databases will be searched from their inception to March 2021. Languages will be restricted to English and Chinese. The search strategy will be formed using the following key phrases: “Bailing capsules,” “pulmonary disease,” “chronic obstructive pulmonary disease,” “randomized controlled trial,” “randomised controlled trial,” randomly∗, and RCT∗.

#### 2.3.2 Searching other resources

The authors will scrutinize the reference lists of each included relevant publication and review article for extra references. An additional search will be conducted on ClinicalTrials.gov (www.ClinicalTrials.gov) to find unpublished data.

### 2.4 Data collection and analysis

#### 2.4.1 Study selection and data extraction

**Figure 1:**
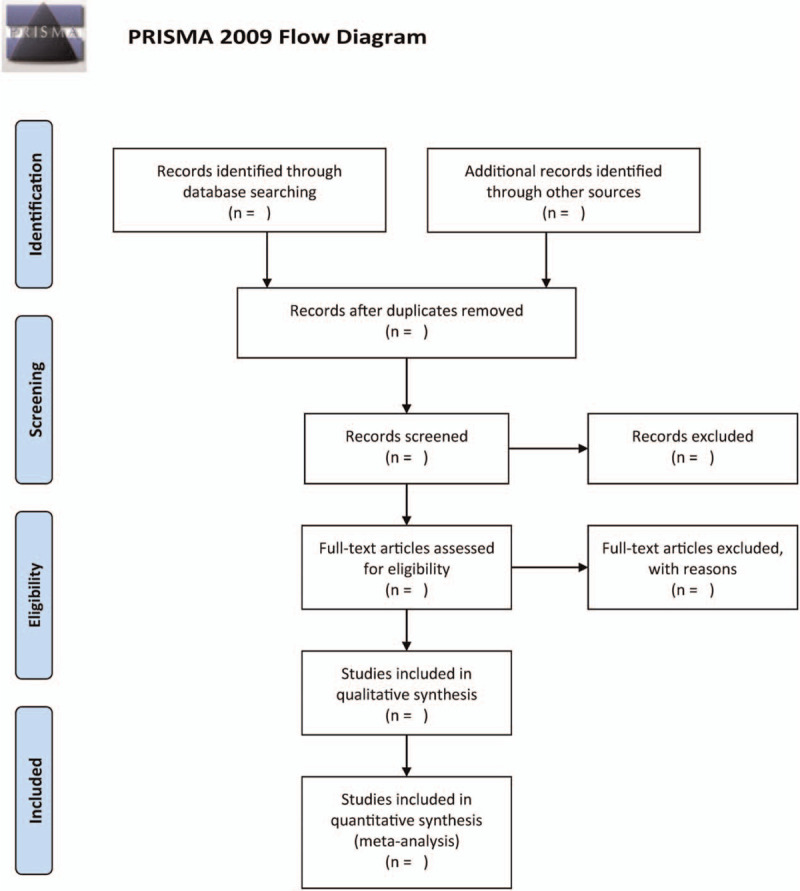
The research flowchart.

Two scholars will independently scrutinize the titles and abstracts of all the obtained studies. Once the studies that do not satisfy the inclusion criteria are excluded, the scholars will go through the full text to determine studies that are likely to satisfy the inclusion criteria to decide on inclusion. The data extraction items include: the first author, publication year, age, gender, number of participants, intervention method, course of treatment, and outcomes. Two investigators will cross-check the results of the included trials, and when differences arise making it difficult to determine whether to include them through discussion or the intervention of a third party, the selection process outlined in Figure 1 is utilized.

#### 2.4.2 Assessment of risk of bias in included studies

Two independent scholars will assess the methodological quality of the included studies, the evaluation is in accordance with the Cochrane Collaboration's Handbook.^[12]^ Any disagreement between scholars is fixed through discussion.

#### 2.4.3 Statistical analysis

The RevMan 5.3 software (Copenhagen) will be employed to conduct all statistical analysis. The risk ratio with 95% confidence interval (CI) will be used to calculate the results of dichotomous variable. The mean differences or standardized mean differences with 95% CI will be used to calculate the results of continuous variable. This study adopts the I^2^ statistic to assess the heterogeneity in the studies included for all analyses. In the event any substantial heterogeneity is identified (I^2^ > 50%), the random-effects model will be used to merge the results. Otherwise, the fixed-effects model will be utilized to merge the results.^[13,14]^ The authors plan to use the sensitivity analysis to assess the stability of our findings. If applicable, the authors will also create and examine funnel plots and Egger tests to examine any bias in the publication.

## 3 Discussion

Recently, the RCTs of Bailing capsules in the treatment of COPD have gradually increased. Published research has demonstrated that the application of Bailing capsules holds a significant position for treating COPD. However, these results are not conclusively proven. Therefore, the present study will be the first review to explore the efficacy and safety of Bailing capsule when used to treat COPD patients. The goal is to provide findings that will aid practitioners make clinical recommendations for COPD patients and help complete clinical decisions to enhance the therapeutic strategy for COPD.

## Author contributions

**Data curation:** Yumiao Zhao, junli jia.

**Formal analysis:** Long Zhang.

**Funding acquisition:** Weili Chu, Qinfu Xu, Yanbing Sheng.

**Investigation:** Yumiao Zhao, Lisha Huang, Qinfu Xu, Aiguo Xu.

**Methodology:** Yumiao Zhao, junli jia, Weili Chu, Aiguo Xu.

**Project administration:** Yumiao Zhao, Lisha Huang, Qinfu Xu, Yanbing Sheng.

**Resources:** junli jia, Weili Chu.

**Software:** Long Zhang, Yanbing Sheng.

**Supervision:** Yumiao Zhao, Lisha Huang, Weili Chu.

**Validation:** Long Zhang, Aiguo Xu.

**Visualization:** Long Zhang.

**Writing – original draft:** Long Zhang.

**Writing – review & editing:** Long Zhang, Aiguo Xu.
